# Time-dependent universal conductance fluctuations in IrO_2_ nanowires

**DOI:** 10.1186/1556-276X-7-673

**Published:** 2012-12-13

**Authors:** Yong-Han Lin, Lu-Yao Wang, Juhn-Jong Lin

**Affiliations:** 1 Institute of Physics, National Chiao Tung University, Hsinchu 30010, Taiwan; 2 Department of Physics, Fu Jen Catholic University, Hsinchuang 24205, Taiwan; 3 Department of Electrophysics, National Chiao Tung University, Hsinchu 30010, Taiwan

**Keywords:** Quantum-interference effect, Universal conductance fluctuation, Mobile defect, Iridium dioxide nanowire, Rutile structure

## Abstract

Single-crystalline iridium dioxide nanowires show the time-dependent universal conductance fluctuations (TUCFs) at cryogenic temperatures. The conductance fluctuations persist up to temperature *T* as high as nearly 10 K. The root-mean-square TUCF magnitudes increase with decreasing *T*, reaching approximately 0.1 *e*^2^ / *h* at 1.7 K. We ascribe these conductance fluctuations to originating from the conduction electrons scattering upon mobile defects (moving scattering centers). Our measured TUCF characteristics are satisfactorily explained in terms of the existing TUCF theory in its three-dimensional form. The extracted electron dephasing length *L*_*φ*_(1.7 K) ≃90 nm is smaller than the diameter (≈ 180 nm) of our nanowires.

## Background

Quantum-interference effects often manifest in the electronic transport properties of miniature conductors at cryogenic temperatures [1,2]. The recent development in nanoscale material synthesis methods has made the fabrications of quasi-one-dimensional (Q1D) nanowires widely accessible. One of the experimental realizations of the marked quantum-interference effects is the observation of the universal conductance fluctuations (UCFs) [1-3] in Q1D metallic [4,5] and heavily doped semiconductor [6,7] nanowires. In sharp contrast to the classical thermal noise, the UCF magnitudes increase with reducing temperature *T*[8-11], owing to the inherent quantum nature of the electron waves traversing in a weakly random potential. In the limit of *T*→0, the root-mean-square UCF magnitudes are theoretically predicted to reach a universal value of *Ce*^2^ / *h*, where the constant *C* depends on sample dimensionality and is of order unity in one, two and three dimensions. A weakly random potential realized in a given sample corresponds to a specific impurity configuration. In the case of the presence of static defects alone, magnetic-field (and Fermi-energy, via a back-gate voltage) dependent UCFs can be observed. This kind of aperiodic UCF ‘magneto-fingerprints’ are largely reproducible if the sample is constantly kept at low temperatures and thus, the impurity configuration remains unaltered during the course of the measurement. Such magnetic-field dependent UCFs have been commonly observed in the past three decades [12-15]. The second kind of conductance fluctuations is the time-dependent UCFs (TUCFs) which have rarely been seen in experiments using conventional lithographic metal mesoscopic structures [16-18]. Recently, two of the authors have observed pronounced TUCFs in single-crystalline RuO_2_ nanowires grown by the thermal evaporation method [4]. The TUCF signals persisted up to as high as *T* > 10 K. The measured TUCFs were ascribed to originating from the scattering of conduction electrons with *mobile* defects, i.e., moving scattering centers [19]. A quantitative comparison with the existing theoretical predictions [20,21] was satisfactory and, in particular, the number of mobile defects in a phase-coherent volume had been inferred. The mobile defects were proposed to be associated with certain point defects (e.g., oxygen vacancies) which were contained in the as-grown nanowires.

In this paper, we would like to show that notable TUCFs also exist in single-crystalline iridium dioxide (IrO_2_) nanowires grown by the distinctly different metal-organic chemical vapor deposition (MOCVD) method. Taking together the results obtained in these two complimentary RuO_2_ and IrO_2_ nanowire experiments, we demonstrate that mobile defects are common and rich in conducting metal oxide nanowires with rutile structure, regardless of how the nanowires are synthesized. This observation could have important bearing on the fundamental understanding and future applications of nanoscale metallic oxide materials. We would like to mention that TUCFs in conventional metal mesoscopic structures fabricated by physical deposition in conjunction with lithographic method only occur at sub-kelvin temperatures [16-18].

## Methods

**Figure 1:**
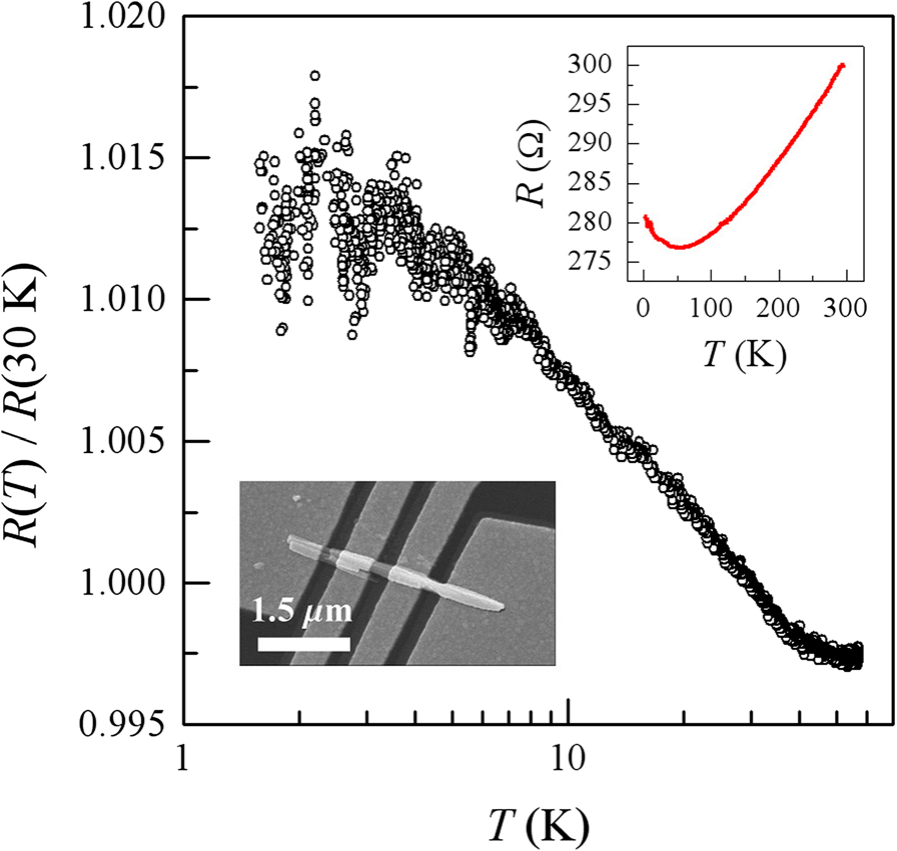
**Variation of normalized resistance *R***(*T*)/*R*** (30 K) with logarithm of temperature for the NW1 device.** The insets show the resistance as a function of temperature between 1.7 and 300 K and an SEM image of the NW1 device taken from [22].

Single-crystalline IrO_2_ nanowires were grown by the MOCVD method. The morphology and atomic structure of the nanowires were studied by scanning electron microscopy (SEM) and transmission electron microscopy. Four-probe single nanowire devices were fabricated by the electron-beam lithography, as described previously [22]. (The inset of Figure 1 shows an SEM image of the NW1 device taken from [22]. In [22], the electrical transport properties of the two NW1 and NW2 nanowire devices had been studied at high temperatures of 30 to 300 K.) The resistance measurements were performed on a standard ^4^He cryostat. A Linear Research LR-700 ac resistance bridge (Linear Research Inc., San Diego, CA, USA) operating at a frequency of 16 Hz was employed for resistance measurements. An excitation current of ≲ 100 nA (so that the voltage drop ≲ *k*_B_*T*/*e*, where *k*_B_ is the Boltzmann constant, and *e* is the electronic charge) was applied to avoid joule heating. Table 1 lists the parameters of the two nanowires studied in this work. 

**Table 1:** **Values of relevant parameters for two IrO**_2_**nanowire devices**

**Device**	***d*(nm)**	***L*(*μ*m)**	***ρ*(300 K) (*μΩ* cm)**	***ρ*(10 K) (*μΩ* cm)**	***D*(cm^2^/s)**	***l*(nm)**
NW1	≈ 180	≈ 0.83	295	270	1.6	0.74
NW2	≈ 180	≈ 0.83	220	185	2.4	1.08

*d* is the diameter, *L* is the voltage probe distance in a four-probe geometry, *D* is the electron diffusion constant, and *ℓ* is the electron mean free path. *D* and *l* are for 10 K.

## Results and discussion

The inset of Figure 1 shows the temperature dependence of resistance for the NW1 device from room temperature down to 1.7 K. This figure clearly reveals that the overall electrical transport property of this single-crystalline nanowire is metallic, as previously established theoretically [23] and experimentally [22,24]. At temperature *T* below about 50 K, the resistance increases slightly with further reduction of *T*, suggesting that this nanowire lies in the weakly disordered regime of *k*_F_*l* > 1, where *k*_F_ is the Fermi wavenumber, and *l* is the electron mean free path (*k*_F_*l* ≈ 6 in this particular nanowire). The small relative resistance increase of *R*(1.7 K) / *R*(50 K) ≃1.014 can arise from the contributions of the weak localization and electron-electron interaction effects [2,25] (and possibly also from other effects such as the two-level tunneling systems [4,26]).

The main panel of Figure 1 plots the normalized resistance, *R*(*T*) / *R*(30 K), as a function of temperature for *T* < 60 K. The nanowire resistance was recorded while the temperature was decreased relatively slowly. What is most interesting in this figure is the notably increased resistance distribution at a fixed *T* as the temperature is lowered to approximately below 6 K. As shown in the previous studies [4,16-18], this increased resistance distribution with decreasing temperature directly manifests the TUCF behavior whose origin is the existence of moving scattering centers in this particular nanowire. As a consequence, the measured resistance is a function of time at a given temperature. This observation comprises the central theme of this paper. This resistance distribution reflects the presence of the TUCF phenomenon. Thus, we shall argue in our succeeding discussion that a small fraction of the point defects contained in our as-grown nanowires must be the mobile defects. It should be noted that the TUCFs are originated from an inherent quantum-interference mechanism. Contrary to the thermal noise, the fluctuation magnitudes of the TUCFs progressively diminish as the temperature increases.

**Figure 2:**
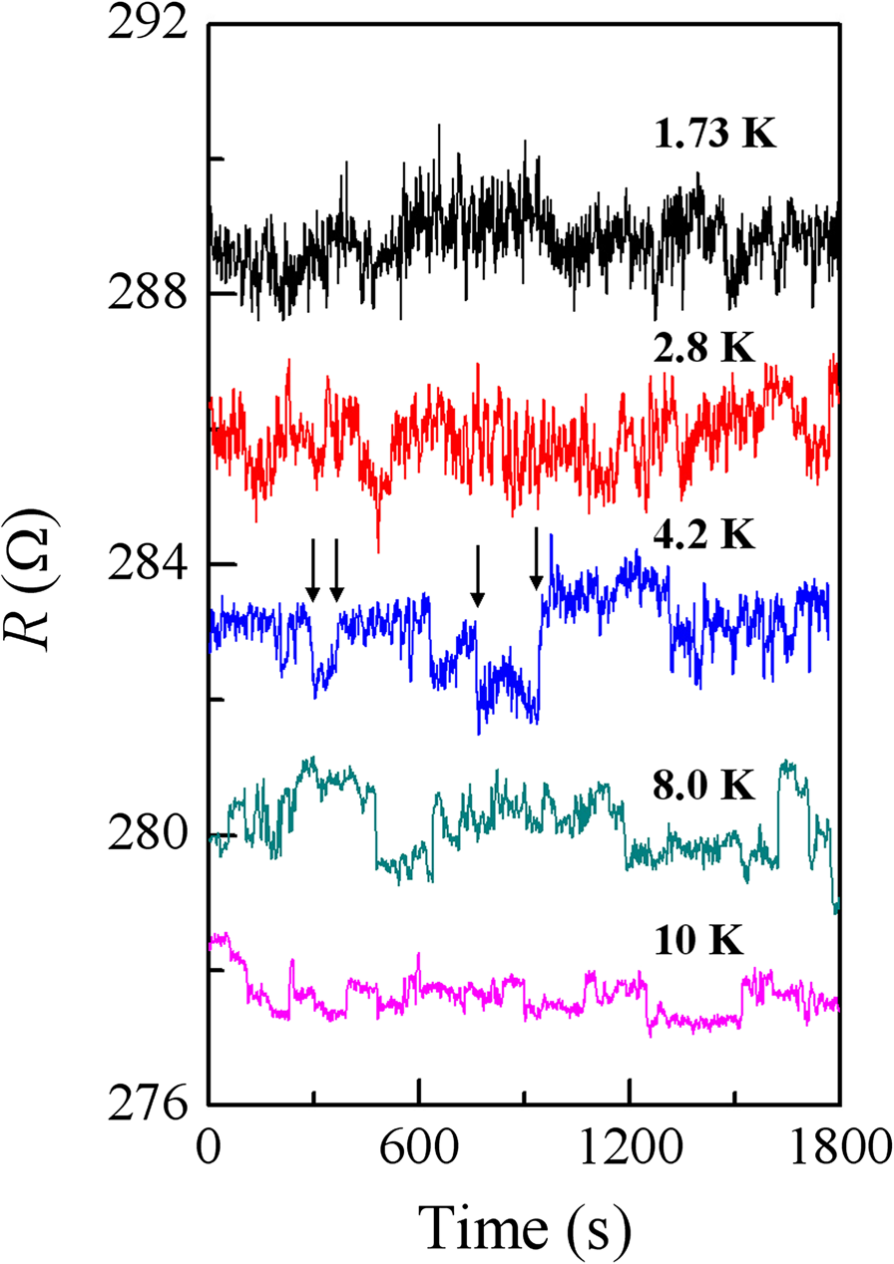
**Variation of resistance with time for the NW1 device.** Resistance as a function of time for the NW1 device at five temperatures, as indicated. The resistances at 8.0, 4.2, 2.8, and 1.73 K have been offset by 2, 4, 6, and 8 *Ω*, respectively, for clarity. The arrows indicate four slow fluctuations occurring at ≈ 280, ≈ 370, ≈ 760, and ≈ 945 s in the resistance curve for *T* = 4.2 K.

We discuss the TUCF features in a more intuitive manner. In Figure 2, we plot the resistance as a function of time for the NW1 device at several *T* values below 10 K. Inspection of this figure clearly indicates that, at a given *T*, the resistance fluctuates with time. There exist both overlapping fast fluctuations and individual slow fluctuations (the slow ones are indicated by arrows in some of them). In particular, the magnitudes of the fast fluctuations increase with decreasing temperature. As the temperature increases to about 10 K, the fast fluctuations disappear to within our experimental error (i.e., our instrumental noise), while the slow fluctuations mimic random telegraph noise [27]. In order to compare with the existing TUCF theory of Feng [21], we shall focus our discussion on the fast resistance fluctuations in this paper. 

**Figure 3:**
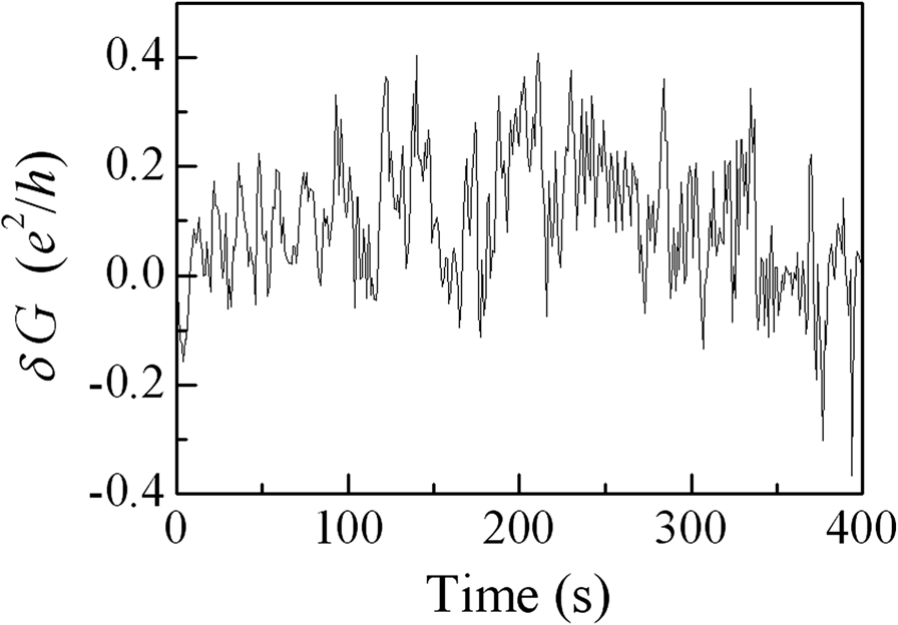
**TUCFs for the NW1 device.** Conductance variation *δG* = *G* − 〈*G*〉 versus time for the NW1 device at *T* = 1.73 K plotted in units of the quantum conductance *e*^2^ / *h*.

Figure 3 plots the temporal variation of the conductance fluctuations *δG* = *G* − 〈*G*〉 in units of the quantum conductance *e*^2^/*h* for the NW1 device at *T* = 1.73 K, where 〈*G*〉 is the measured conductance *G* = 1 / *R*averaged over time. It can be seen that the ‘peak-to-peak’ conductance fluctuation magnitude reaches ≈ 0.1 to 0.2 *e*^2^ / *h*. This result of a fraction of *e*^2^ / *h* at low *T* provides a meaningful indication that our measured conductance fluctuations are associated with the TUCF phenomenon [4,21]. 

**Figure 4:**
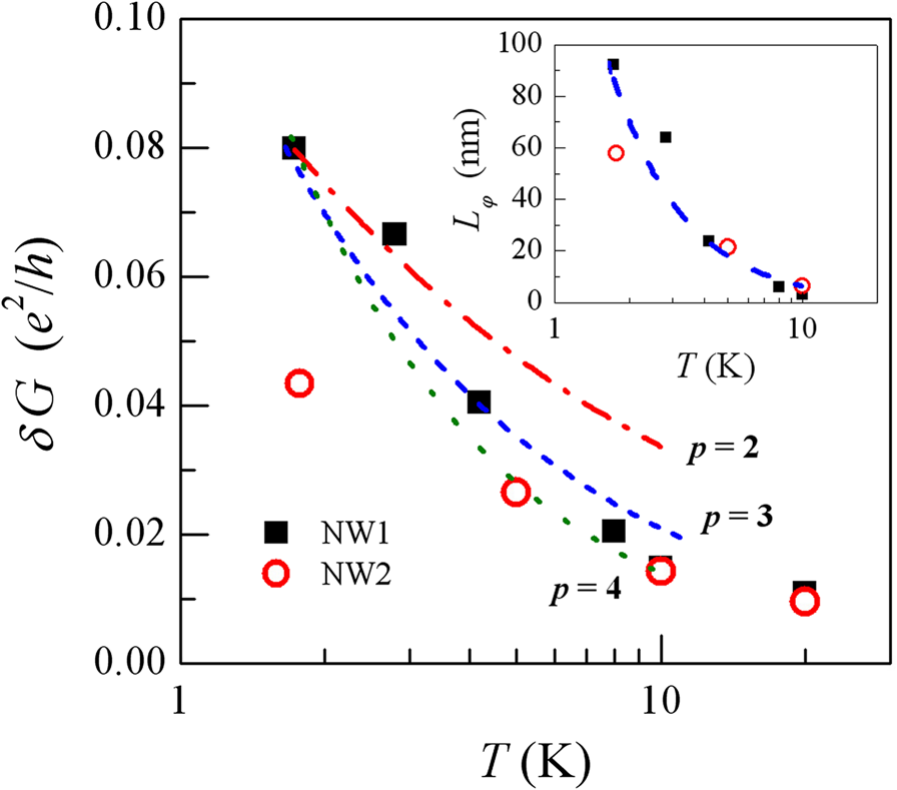
**Root-mean-square conductance fluctuation magnitude ***δG*_rms_**as function of temperature for NW1 and NW2 devices.** The dot-dashed, dashed, and dotted curves are the theoretical predictions of Equation 1 with the exponent of temperature *p* = 2, 3, and 4, respectively, for the electron-phonon relaxation rate 1/*τ*_ep_ = *A*_ep_*T*^*p*^. The inset shows the extracted electron dephasing length *L*_*φ*_ as a function of temperature for the two nanowires. The dashed curve indicates a *T*^3^ temperature dependence. The data points at 10 K could be subject to large uncertainties and are only drawn for reference.

In order to quantitatively analyze the fast TUCFs, we evaluate the root-mean-square magnitude of the conductance fluctuation defined by $\left(\delta G_{\text{rms}}=\sqrt{\langle {\left(G-\langle G\rangle \right)}^{2}\rangle }\right)$, where 〈…〉denotes the averaging over a proper time interval while excluding the slow fluctuations. Figure 4 plots *δG*_rms_ as a function of *T* in double-logarithmic scales for the NW1 and NW2 devices studied in this work. This figure clearly demonstrates that our measured *δG*_rms_increases with decreasing *T* in both nanowires. As *T* increases to about 10 K, the size of *δG*_rms_becomes indistinguishable from the instrumental noise (our instrumental noise level is ≈ 0.01*e*^2^ / *h* in the present study; therefore, the data points at 20 K in Figure 4 are only plotted for reference).

Theoretically, the TUCFs in different sample dimensionalities and under different conditions have been studied by Al’tshuler [8,9] and Lee, Stone, and Fukuyama [10,11]. In particular, Feng and coworkers [20,21] have proposed that the TUCFs are very sensitive to the motion of single or a few mobile defects. In order to interpret our TUCF data, it is important to identify the effective sample dimensionality of our IrO_2_ nanowires. In the quantum-interference studies, the effective nanowire dimensionality is determined by the ratio of the electron dephasing length *L*_*φ*_ to the nanowire diameter *d*. A nanowire lies in the Q1D regime if *L*_*φ*_ / *d* ≫ 1, and in the three-dimensional (3D) regime if *L*_*φ*_ / *d* ≪ 1. We have found that our measured *δG*_rms_(*T*) definitely cannot be consistently described by the Q1D form of the TUCF theory, because using the Q1D form would always lead to an extracted *L*_*φ*_ smaller than *d*. Therefore, we have turned to compare our results with the 3D theoretical form. Feng predicted that for a 3D sample and in the ‘saturated’ regime, the TUCF magnitudes are given by [21]

(1) $${\left(\delta G_{\text{rms}}\right)}^{2}=28.2\langle G^{2}\rangle \frac{1}{k_{F}^{4}\ell^{2}}\frac{L_{\varphi }}{Ld^{2}}\;,$$

where *L* (>*L*_*φ*_) is the nanowire sample length. In the theory, the so-called saturated regime refers to the regime with the parameter *β* ≫ (*ℓ*/*L*_*φ*_)^2^, where *β* stands for the ratio of the number of mobile defects to the number of total (static and mobile) defects.

We have carried out least-squares fits of our measured *δG*_rms_(*T*) to the predictions of Equation 1, using $\left(L_{\varphi }=\sqrt{D\tau_{\varphi }}\right)$ as the sole adjustable parameter, where *D* is the electron diffusion constant, and *τ*_*φ*_ is the *T* dependent electron dephasing time. Explicitly, in a 3D weakly disordered conductor, the total electron dephasing rate is essentially given by two contributions [28,29]: $\left(1/\tau_{\varphi }=1/\tau_{\varphi }^{0}+1/\tau_{\text{ep}}\right)$, where the first contribution $\left(1/\tau_{\varphi }^{0}\right)$ is a constant or a very weakly *T*-dependent term [30,31], and the second contribution $\left(1/\tau_{\text{ep}}=A_{\text{ep}}T^{p}\right)$ denotes the electron-phonon relaxation rate, with *A*_ep_ being the electron-phonon coupling strength, and *p* being an exponent of temperature. In general, the value of *p* depends on the measurement temperature interval as well as the degree of disorder in the sample. Typically, 2 ≤ *p* ≤ 4 in 3D metals [30-32].

Since the NW1 and NW2 devices reveal overall similar TUCF features (except that the TUCF magnitudes in the latter are somewhat smaller than those in the former), we shall concentrate the following discussion on the NW1 device. Our fitted results with the exponent of temperature *p* being fixed to be 2, 3, or 4 are shown by the dot-dashed, dashed, and dotted curves, respectively, in the main panel of Figure 4 (for simplicity, we chose the value of *p* to be an integer in our least-squares fits to Equation 1). Inspection of this figure indicates that Equation 1 can satisfactorily describe the experimental results. The fit with the exponent *p* = 3 gives a slightly (notably) better description than that with *p* = 4(2). Numerically, our fitted values of the relevant parameters for the NW1 device are listed in Table 2. The extracted electron-phonon coupling strength *A*_ep_ ≈ 4 × 10^9^ K^−*p*^ s^−1^ is compatible to that previously found in normal metals, such as RuO_2_ nanowires [4] and AuPd wires and films [28,29]. We would like to note in passing that a more quantitative extraction of the value of *p* would require further measurements on, e.g., the magnetoresistances in the weak-localization effect [25,28,29]. 

**Table 2:** **Values of fitted parameters for the electron dephasing rate**$\left(1/\tau_{\varphi }\;=\;1/\tau_{\varphi }^{0}\;+\;A_{\text{ep}}T^{p}\right)$**in NW1 device**

**Exponent *p***	$\left(1/\tau_{\varphi }^{0}\right)$**(s^−1^)**	***A*_ep_ (K^−*p*^ s ^−1^)**
2	≈ 1×10^9^	≈ 6×10^9^
3	≈ 7×10^8^	≈ 4×10^9^
4	≈ 5×10^8^	≈ 2×10^9^

Our extracted *L*_*φ*_ values at different temperatures between 1.7 and 10 K are plotted in the inset of Figure 4. We obtain a relatively short *L*_*φ*_(1.7 K) ≈ 90 nm in the NW1 device. Moreover, *L*_*φ*_* decreases rapidly with increasing****T*, reaching a small size of***L*_*φ*_(8 K) ≈ 10 nm. The extracted relatively short *L*_*φ*_ values may partly arise from non-negligible experimental uncertainties. First, our measured TUCF magnitudes are small, which render large uncertainties in the separation and evaluations of *δG*_rms_ from the background instrumental noise (experimentally, our TUCF signals become hardly distinguished from the instrumental noise as *T* ≳ 8K). Second, our nanowires with diameters of 180 nm may fall close to the 1D-to-3D crossover regime with regards to the quantum-interference effects, instead of falling deep in the 3D regime. Therefore, Equation 1 is probably only about to become fully valid (however, we would like to remind that our data definitely cannot be described by the 1D form of TUCF theory). Third, the determination of the relevant sample volume *Ld*^2^ is subject to some uncertainties. In any case, note that we obtain *L*_*φ*_ < *d* over our measurement *T* range; hence, the 3D TUCF phenomenon in this NW1 device is more or less justified.

## Conclusions

We have observed TUCFs at cryogenic temperatures in metallic single-crystalline IrO_2_ nanowires grown by the MOCVD method. The TUCFs originate from the scattering of conduction electrons upon mobile defects. Our measured TUCF magnitudes as a function of temperature are satisfactorily described by the existing theory in the three-dimensional regime. Taken together with our previous observations in single-crystalline RuO_2_ nanowires grown by the distinctly different thermal evaporation method [4], the present study indicates that moving scattering centers may be common to the conducting metal oxide rutile nanostructures, regardless of how they are synthesized. Our observations could have important bearing on the fundamental research and technological applications of synthetic metal oxide nanoelectronic devices.

## Competing interests

The authors declare that they have no competing interests.

## Authors’ contributions

YHL fabricated the devices and conducted the electrical measurements. LYW analyzed the results and wrote the manuscript. JJL coordinated and supervised the overall study and helped to draft the manuscript. All authors read and approved the final manuscript.
